# Exploring Esketamine's Therapeutic Outcomes as an FDA-Designated Breakthrough for Treatment-Resistant Depression and Major Depressive Disorder With Suicidal Intent: A Narrative Review

**DOI:** 10.7759/cureus.53987

**Published:** 2024-02-10

**Authors:** Suneeta Kumari, Hassan A Chaudhry, Adam Sagot, Stacy Doumas, Hussain Abdullah, Eric Alcera, Ramon Solhkhah, Saba Afzal

**Affiliations:** 1 Psychiatry, Hackensack Meridian Ocean Medical Center, Brick, USA; 2 Medical School, Medical University of Lublin, Lublin, POL; 3 Psychiatry and Behavioral Sciences, Youth Neuroscience Organization, Tbilisi, GEO; 4 Psychiatry, Northshore University Health System, Evanston, USA

**Keywords:** fda approval, placebo controlled trials, treatment-related adverse event, suicide prevention, suicide and depression, montgomery-asberg depression rating scale, nmda receptor antagonist, selective serotonin reuptake inhibitor (ssri), esketamine, treatment-resistant depression

## Abstract

The expansive spectrum of major depressive disorder (MDD) continues to pose challenges for psychiatrists to treat effectively. Oral antidepressant (OAD) medications that alter monoamine neurotransmitters, mainly selective serotonin reuptake inhibitors (SSRIs) and selective norepinephrine reuptake inhibitors (SNRIs), have been the mainstay of therapy for decades. Although these drugs have been largely beneficial, a considerable subset of patients do not respond adequately to multiple conventional therapies administered for an appropriate length of time, leading to a diagnosis of treatment-resistant depression (TRD).

Ketamine, a non-monoaminergic drug, has long been known for its beneficial effects on TRD when given intravenously (IV). Between 2019 and 2020, an intranasal formulation of the S (+) enantiomer of racemic ketamine, esketamine (ESK), was granted “breakthrough designation" by the FDA and approved for the indications of TRD and MDD patients exhibiting acute suicidal intent.

The objective of this narrative review was to review the academic literature and collect clinical evidence that may corroborate intranasal ESK's effectiveness for its approved indications while addressing its safety and tolerability profile, adverse effects, and impact on cognition. An overview of the drug's origins, pharmacology, and standard treatment regimen are provided. The outcomes from double-blinded randomized control trials (DB-RCTs) of ESK are outlined to demonstrate the efficacy and safety data leading to its FDA approval, along with its long-term post-market safety outcomes. Comparative trials between ESK and ketamine are then evaluated to highlight ESK's consideration as a more practical alternative to ketamine in common clinical practice. The authors further discuss currently approved and developing therapies for TRD, propose future research directions, and identify the inherent limitations of the review and further research.

To conduct the research required, three digital databases (PubMed, Medline, and ClinicalTrials.gov) were queried to search for key terms, including *ketamine, esketamine, treatment-resistant depression, and biomarkers, *using automation tools* *along with selective search engine results*.* After streamlining the results by title and abstract and removing duplicates, a total of 37 results were chosen, of which 18 are clinical trials. A reduction in the Montgomery-Asberg Depression Rating Scale (MADRS) score was the primary efficacy endpoint for most of these clinical trials.

In conclusion, intranasal ESK, when used as an adjunct to market OADs, shows greater efficacy in treating TRD and MDD with suicidal intent compared to OADs and placebo alone and provides a more suitable alternative to IV ketamine. It is important to note that further research is required to fully understand the novel mechanism of action of ESK, as well as the establishment of a consensus definition of TRD, which may facilitate better detection and treatment protocols. More focused quantitative and qualitative ESK studies are needed, as well as those pertaining to its use in patients with co-existing mental illnesses.

## Introduction and background

Major Depressive Disorder (MDD) is one of the most prevalent, chronic, and disabling psychiatric disorders, which can severely diminish the psychosocial functioning and quality of life of those affected. The World Health Organization reports through the Institute of Health Metrics and Evaluation that approximately 280 million people globally have depression [1]. This is an estimated 3.8% of the global population experiencing depression, including four percent of adults, with women being more affected, with six percent of women vs. four percent of men being affected [1]. These rates include 5.7% of adults older than 60 years [1]. Significantly, approximately 700,000 people die each year by suicide [2]. Despite effective treatments, more than 75% of people in low- and middle-income countries do not receive treatment [2]. Although no universally accepted definition for treatment-resistant depression (TRD) currently exists, the United States (US) Food and Drug Administration (FDA) as well as the European Medicines Agency (EMA) have adopted the most common definition of TRD being defined in MDD patients that lack an adequate response to two or more antidepressants given at sufficient dosage and treatment duration, with patient adherence [3]. In most instances of TRD, patients have exhausted numerous therapeutic options, including selective serotonin reuptake inhibitors (SSRIs), selective norepinephrine reuptake inhibitors (SNRIs), tricyclic antidepressants, and adjunctive therapy. Yet, it is estimated that 30% of patients remain unresponsive to treatment [3], presumably due to multiple sociodemographic, clinical, treatment, and contextual factors [3]. However, very few of these differences are reliably predictive of patient non‐response across various types of treatment [3]. Subsequently, this has created an urgent and unmet need for the development of innovative TRD therapies. Intravenous (IV) ketamine has long been known and tested for its beneficial effects in TRD [3], but in the past decade in particular, an increased interest has accumulated in esketamine (ESK), a psychedelic drug related to ketamine for treating TRD. Clinical trial results from former and ongoing studies have shown promising results, suggesting that ESK may serve as an effective add-on treatment option for TRD patients [4]. With its novel mechanism of action (MOA) and rapid onset, ESK has the potential to assist in reducing refractory depressive symptoms, solving an unmet need for patients with TRD [4].

The FDA approved an ESK nasal spray for treating TRD in adults in March 2019 under the brand name Spravato®, produced by Janssen Pharmaceutical Company [4]. In 2020, a supplemental indication was added for MDD patients displaying acute suicidal intent [4]. Due to its risk for abuse and diversion, ESK is classified as a Schedule III (CIII) controlled substance [4]. Patients with a history of substance use disorder (SUD) should be carefully assessed before being initiated on ESK, and continually monitoring patients for signs of substance misuse is critical [4]. To address these hurdles, ESK is only available through a regulated distribution system under the Spravato® Risk Evaluation and Mitigation Strategy (REMS) program, which includes a list of officially registered patients and specific healthcare sites [4]. ESK should only be administered to patients within licensed, certified healthcare settings if both patients and sites are enrolled in the REMS program [4]. Furthermore, due to the risk of adverse effects such as hypertension, sedation, and potential dissociative effects after administration, enrolled patients should receive ESK under the direct supervision of a healthcare provider and then be monitored for a minimum of two hours post-treatment [4]. The utilization of ESK in pediatric patients has not currently been authorized in pediatric populations [4].

The objective of this narrative review was to review the academic literature and collect clinical evidence that may corroborate intranasal ESK's effectiveness for its approved indications while addressing its safety and tolerability profile, adverse effects, and impact on cognition. An overview of the drug's origins, pharmacology, and standard treatment regimen are provided. Then outcomes from double-blinded randomized control trials (DB-RCTs) of ESK are outlined to demonstrate the efficacy and safety data leading to its FDA approval, along with its long-term post-market safety outcomes. Comparative studies between ESK and ketamine are then provided and analyzed to highlight ESK's consideration as a more practical alternative to ketamine in common clinical practice. The authors further discuss currently approved and developing therapies for TRD, propose future research directions, and identify the inherent limitations of this review and further TRD research.

For the purpose of this review, the digital databases PubMed, Medline, and ClinicalTrials [38] were queried, and the results were limited to the years 2010-current. Key search terms included a mix of the following: ketamine, esketamine, depression, treatment-resistant depression, and biomarkers. The results were run with and without Boolean operators "AND/OR" in between terms and with and without the key filter: clinical trials. The results yielded 91 results for clinical trials, of which 18 trials have been chosen, and 27 results for biomarkers, of which two were sufficiently chosen. After streamlining by title and abstract, removing duplicates, and supplementing with selective search engine results, a total of 37 references have been analyzed to compose this narrative review, including 18 RCTs. Of the 18 RCT's, 13 involve intranasal ESK directly, with two involving IV ESK, two independent ketamine trials, including an efficacy trial, and a pilot study from the year 2000 from the related articles tab for referencing purposes. One comparability study has been added to negate the bias of functional unblinding. Additionally, 16 other entries have been chosen, including a post-hoc analysis, an advisory panel summary, four webpages, a mix of several meta-analyses/systematic reviews, and topic-specific publications. ClinicalTrials was consulted simultaneously alongside published trial results, using corresponding trial registration numbers to extract relevant data where deemed necessary.

Historical overview of ESK

Phencyclidine (PCP) is thought to have been the foundational substance that enabled the formation of ketamine and, later, its S(+) enantiomer, or ESK. Organic chemists at the Parke Davis Company produced PCP under the trade name Sernyl for the first time in 1956 [5,6]. PCP was seen as capable of triggering rodents, dogs, pigeons, and monkeys into inebriated, hallucinogenic, and/or catatonic states [7]. Although PCP was demonstrated to be a safe and reliable anesthetic option for humans, it caused patients to develop prolonged and severe delirium, making its use undesirable [5,6]. Subsequently, numerous attempts were made to synthesize a shorter-acting version of PCP that would have the same anesthetic potency and effects while causing patients to experience significantly less delirium [5-7].

Calvin Stevens, an organic chemist and consultant to Parke Davis Company, ultimately developed ketamine, designated CI-581, in 1962, forming a water-soluble PCP derivative [5,6]. The ketamine molecule is made up of an asymmetric carbon atom with two enantiomers: the S(+) isomer and the R(−) isomer [6]. Ketamine, a structural analog of PCP with one-tenth the potency of PCP, was ultimately chosen for human trials [5]. On August 3, 1964, at the University of Michigan, Dr. Edward Domino and Dr. Guenther Corssen, professors of pharmacology and anesthesiology, respectively, administered the first human dose to inmates at a prison [5,6]. Dr. Domino and Dr. Corssen's first study of ketamine's effects on 20 patients showed that it was safe and effective as a clinical anesthetic and a great alternative to PCP [5,6]. The pair published the first clinical study of ketamine in 1966, claiming it could be used as a human anesthetic [5,6]. Ketamine users often experience "dissociative anesthesia," which describes the distinctive state where patients appear awake with preserved airway reflexes and respiratory drive but are unable to react to sensory input [6]. The reputability of ketamine as a remarkably safe anesthetic makes it potentially useful for diverse patient populations [7].

Ketamine was approved for veterinary use as early as 1963 in Belgium and became available as a prescription in the United States by 1969 in the form of ketamine hydrochloride [5,6]. It was FDA-approved for human consumption in 1970 under the brand name Ketalar [5,6]. It was referred to as a "rapidly acting, non-barbiturate general anesthetic" recommended for brief procedures [5-7]. In addition to being a staple in veterinary medicine, it has demonstrated numerous clinical applications in human medicine, particularly in pain management and anesthesia [5-7]. Moreover, it has shown anti-inflammatory, anti-tumor, and neuroprotective properties [5-7]. It is believed that ketamine acts as a non-competitive N-methyl-D-aspartate (NMDA) antagonist and increases the excitatory neurotransmitter glutamate, which may increase structural synaptic connectivity, allowing for it to work as an antidepressant with prolonged effects [5-7].

ESK pharmacodynamics and pharmacokinetics

ESK, the (S)+enantiomer of ketamine, demonstrates a 2-4 times higher affinity for NMDA receptors than its isomer R(-) ketamine [7,8]. It acts as an NMDA receptor antagonist in the same way that ketamine does, which can affect fast excitatory glutamate transmission, increase brain-derived neurotrophic factor (BDNF) release, and stimulate synaptogenesis [7,8]. It also has local anesthetic effects and acts on opioid, cholinergic, monoamine, purinergic receptors, and adrenoreceptor systems [7,8]. However, the exact mechanism of action of the serotonin and norepinephrine systems as antidepressants is unknown. A standard ESK treatment regimen with pre- and post-treatment protocols, as well as ESK drug-specific information such as pharmacologic properties, standard cost, black box warning, side effects, and contraindications, can be found below in Table 1, as derived from the Spravato Medication Guide [4]. 

**Table 1:** Esketamine treatment protocol & pharmacologic properties Hx: history, mg: milligram, UTI: urinary tract infection, MC: most common, S/E: side effects; C/I: contraindication, BP: blood pressure, AVM: arteriovenous malformation, ICH: intracerebral hemorrhage

Treatment regimen		Route of Administration	Nasal spray
Starting dose	26mg	Absorption	Through the nasal mucosa
Followed by	56mg or 84mg	Self-administration	Yes, under supervision at certified medical facilities/offices
Frequency	Two times per week for four weeks, then one time per week for four weeks, then one time per two weeks for four weeks	Dosage	28mg metered dose delivered in two sprays, one in each nostril
Bioavailability	48%	Time to maximum plasma concentration after last dose	20-40 minutes
Half-lives	7 to 12 hours	Allowance for additional doses	Allow 5 minutes before administering the next device
Continuation of treatment	To be determined by the provider and patient to continue treatment for more than 6-12	Pre-treatment monitoring	Monitor blood pressure and vital signs
Missed treatments	Patient needs to be reassessed and started on different dose or frequency of medication	Post-treatment monitoring	Monitor at least at the 40-minute mark and the 1 hour 30-minute mark post administration. Monitor for at least 2 hours at the medical office
Cost	Estimated to be $590 to $885 per treatment session	Pre-administration restrictions	No eating for 2 hours and drinking for 30 minutes before administration
Boxed warnings	Sedation, dissociation, suicidal ideation, attention, judgment, reaction speed, and motor skills may be impaired	Post-administration restrictions	No driving or operating machinery until the next day after restful sleep
Side Effects	MC S/E's were headache, dizziness, nausea, vomiting, vertigo, and sedation. dissociative effects, anxiety, decreased feeling or sensitivity, lethargy, feeling intoxicated, elevated BP, UTI	Contraindications	Hx of the following disorders are C/I to ESK treatment: aneurysms, AVM's, ICH. Allergic reactions. Not approved for use in pediatric population (safety/efficacy unknown) Potential teratogenicity: may cause embryo/ fetal harm. Breastfeeding while on ESK not recommended

## Review

Esketamine clinical trials

Daly et al. (2018) published the very first proof-of-concept trial, a double-blinded randomized control trial (DB-RCT), to evaluate intranasal ESK's efficacy, safety, and dose dependency [9]. The study employed 4 phases: i) screening, in which 126 were assessed for failure of response to more than one oral antidepressant (OAD), and 67 were selected to participate; ii) double-blind treatment between days 1-15, broken into two one-week periods, iii) optional open-label treatment from days 15-74, and iv) post-treatment follow-up at eight weeks [9]. The blinded component of the study revealed that the least squares (LS) means difference for periods one and two was -4.2 [standard error (SE=2.09, p = 0.02] for the 28 mg dose, -6.3 (SE = 2.07, p = 0.001) for the 56 mg dose, and -9.0 (SE = 2.13, p < 0.001) for the 84 mg dose [9]. This study would become an endorsement of the potential of ESK to treat TRD and the need for clinical trials with larger sample sizes [9]. 

An overview of the most important and relevant ESK clinical trials, including their title, design, results, and determinations, can be seen in Table 2 . The clinical trials listed provide crucial information regarding ESK's tested efficacy and safety outcomes both before and after FDA approval, its comparative trials to ketamine to establish it as a more practical alternative to ketamine, as well as clinical trials to gauge if and to what extent ESK affects cognition. The following summary (Table 2) is followed by a discussion of each of the trials and their respective conclusions.

**Table 2:** Relevant published clinical trials on esketamine Tx: Treatment, DB: Double-blinded; RCT: Randomized control trial; ESK: Esketamine; MADRS: Montgomery-Åsberg depression rating scale; IV: Intravenous; LS: Least squares; SE: Standard error; CI: Confidence interval; SD: Standard deviation, HR: Hazard ratio; NNT: Number needed to treat; IND: Induction; OP: Optimization, MAINT: Maintenance; OAD: Oral antidepressant; SDLP: Standard deviation of lateral position; ANCOVA: Analysis of covariance; CGISS-r: Clinical global impression of severity of suicidality-revised version; SIBAT: Suicide ideation behavior assessment tool; PHQ-9= Personal health questionnaire-9; CGI-S: Clinical global impression of severity,  CADSS: Clinician-administered dissociative states scale, BPRS: Brief psychiatric rating scale

Clinical Trial	Objective	Methods	Results	Conclusions and Future Perspectives
Daly et al. (2018) #NCT01998958 [9]	Phase II, DB, delayed start, RCT: To evaluate the effectiveness of ESK in a cohort of TRD adults	Four-phased, two-period study: i. screening; ii. DB-Tx phase; iii. optional open-label Tx; iv. post-Tx. follow-up. Intranasal ESK administered to 67 screened pts. as an adjunct Tx to OADs. DB-phase: pts. given either a placebo or ESK (24, 56, and 84 mg) twice weekly. In the second period, participants with harsher symptoms were re-randomized between four Tx regimens. Those with milder symptoms kept receiving a placebo. In the open-label phase, the dosage frequency was halved from twice weekly to weekly, and then to every two weeks.	The primary efficacy endpoint was the MADRS score change from the start to the 8^th^ day. of the intervention. Change from baseline in the MADRS total score much higher in all three ESK groups vs. placebo group after 1-week of Tx. Other measurements: CADSS, BPRS	In this first-ever study of intranasal ESK looking at efficacy, safety, and dose response, results indicated that ESK was efficacious in reducing depressive symptoms with rapid onset and sustained response in TRD pts. for up to two months. These findings implied that ESK was a potential therapy, and further research with a larger sample size was required.
Fedgchin et al. (2017) TRANSFORM-1 #NCT02417064 [10]	Phase III, DB, RCT: Evaluation of efficacy and safety of fixed-dose ESK+ OAD regimen	Three phases: 4-week screening/ prospective observation phase 4-week DB Tx. phase Up to 24-week follow-up phase. Randomization of 346 adults with TRD in a 1:1:1: ratio of either (56mg or 84 mg ESK) + OAD twice weekly, vs. placebo + OAD. MADRS score change after 28 days by blinded raters employed as the primary endpoint.	The difference in LS means between ESK 84 mg/OAD vs. placebo control group was -3.2. To evaluate ESK 56 mg, statistical significance for ESK 84 mg was required: hence leading to an inadequate evaluation of ESK 56 mg. The LS mean difference for ESK 56 mg/OAD vs placebo control group was -4.1.	Despite not reaching statistical significance, the Tx effect in both E5K (56/84mg) groups was clinically meaningful. ESK and currently approved OADs had similar changes in depressive symptoms (i.e., MADRS score changes), and safety profiles, with no dose-related safety issues. Overall, the study endorsed the potential safety and efficacy of ESK.
Popova et al. (2019) TRANSFORM-2 #NCT02418585 [11]	Phase III, DB, RCT: Evaluation of flexibly dosed ESK + OAD regimen	Flexible dosed ESK (56 mg or 84 mg) +OAD measured vs. placebo control group using 223 pts. with 114 ESK test participants and 109 in the control group. All participants were given respective therapies twice weekly for four weeks. Follow up at 24 weeks or entry into SUSTAIN-1.	A total of 227 pts. were randomized to Tx regimens with 197 finishing the 28-day therapy trial period. The primary endpoint was the MADRS change between the baseline and the 28th day. More profound changes in the primary endpoint within the ESK group vs placebo group at day 28 (LS means difference = -4.0).	A safety assessment showed the most common side effect was dizziness, alongside vertigo, and dissociation. These were seen more in the ESK group but were transient, mild in nature, and deemed tolerable. Ultimately, therapy with ESK as an adjunctive therapy to an OAD led to meaningful clinical improvements and supported the efficacy of ESK. Safety testing needed more testing.
Ochs-Ross et al. (2020) TRANSFORM-3 #NCT02422186 [12]	Phase III, DB, RCT: Assessing the efficacy of ESK in elderly pts. with MDD (typically worse prognostically)	Evaluated ESK in elderly pts. (≥65 years) using the same testing protocol as TRANSFORM 1-2. MADRS changes on the 28th day were the primary efficacy endpoint. An analysis between the age groups themselves (65–74 vs. ≥75 years) and post hoc analyses, including the age of depression onset was added later.	Esketamine/antidepressant did not achieve statistical significance for the primary endpoint. Greater differences between treatment arms were seen for younger patients (65–74 years) and patients with earlier onset of depression (<55 years). Patients above ≥75 did not show meaningful improvements.	This was a very interesting study in that it showed discrepancies relevant to patient age and age of onset of depression, as considerable variables to effective therapy. As an overall result, analyzing all age groups within the subsets, ESK groups showed a reduction in MADRS score by 3.6 points. Compared to the typical two-three-point decrease seen with currently marketed OADs, ESK was clinically meaningful in combatting depressive symptoms compared to control groups.
Daly et al. (2019) SUSTAIN-1 #NCT02493868 [13]	Phase III, DB, RCT: Assessment of ESK's efficacy in conjunction with an OAD in delaying relapse of a depressive episode in stable (in remission or responsive) TRD pts.	A withdrawal study was conducted between October 2015 to February 2018, enrolling 705 adults with confirmed TRD; 455 entered the OP phase and were treated with ESK (56 or 84 mg) + OAD. After four months (16 weeks), 297 achieved stable remission or response and were entered into the randomized withdrawal phase.	Stable remitters and stable responders were randomized 1:1 to continue ESK or discontinue ESK and started on a placebo (both adjunctive to an OAD). Among the 297 adults, 176 achieved stable remission: 24 in the ESK group and 39 in the placebo group relapsed, or 26.7% vs 45.3%, respectively. OF the 121 stable responders, 16 from the ESK group and 34 from the placebo group relapsed, or 25.8% vs 57.6%, respectively.	Transient loss of taste, vertigo, dissociation, somnolence, and dizziness were reported in greater numbers in the ESK study pts.. Crucially, however, the ESK trial group saw on average a 51% decrease in relapse rates ([HR], 0.49; 95% CI, 0.29-0.84) in stable remitters and 70% in stable responders. pts. who received ESK. This study demonstrated that ESK can both facilitate maintenance as well as significantly reduce relapse in both stable remission and stable response.
Wajs et al. (2020) SUSTAIN-2 #NCT02497287 [14]	Phase III, DB, RCT: To evaluate the long-term safety and efficacy of ESK.	Long-term (up to 1 year) study between October 2015 and October 2017. Direct enrollment of pts. ≥18 years and older or transferred from a short-term study (mainly pts. ≥ 65).	Of 802 enrolled pts., 580 entered & completed the IND phase, while 150 entered and completed the OP/MAINT phase. The most documented TEAEs were dizziness in almost exactly 1/3 of pts., followed by dissociation, nausea, and headache in approximately 1/4th of pts., respectively. Although 76 pts. discontinued the study due to TEAEs, only 55 pts., or 6.9% of participants complained of serious/severe TEAEs. MADRS score reduction during the IND phase lasted during the OP/MAINT phase (mean [SD] change from baseline of respective phase to endpoint: IND, −16.4 [8.76]; OP/MAINT, 0.3 [8.12]).	Most TEAEs were related to dosing and occurred after Tx. Symptoms were typically mild and transient. Death of two pts. unrelated to ESK. Cognition generally enhanced or stayed stable compared to baseline. Dissociative symptoms, which is a concern of ESK therapy, mostly resolve within 1-2 hours. The study demonstrates that a newly initiated OAD in conjunction with long-term ESK nasal spray shows a tolerable safety profile and persistent benefits in reductions in depressive symptoms.
Zaki et al. (2023) SUSTAIN-3 #NCT02782104 [15]	Phase III, DB, RCT: Participants in any of the six phase III, "parent" studies of ESK were enrolled into either a 4-week induction phase or a long-term OP/MAINT phase of SUSTAIN-3. The participants engaged in self-administration of ESK on a biweekly basis for 4 weeks. The dosing frequency was adjusted according to the severity (marker: CGI-S-based algorithm.)	MADRS score reduction and PHQ-9-item were used to assess depressive symptoms of psychosocial disability and to further determine Tx session frequency.	MADRS, PHQ-9 improvements seen. Of 1148 adult pts. with TRD, 458 were enrolled in the IND phase, 420 (91.7%) went on to the OP/MAINT phase, while 342 (30.8%) dropped out for a variety of reasons. However, no clear pattern emerged relative to discontinuation frequency across the trial period.	The safety profile of ESK, with intermittent dosing for up to 4.5 years in SUSTAIN-3 (2,769 cumulative patient-years) was consistent with earlier studies. TEAEs were reported to be mild and transient (headache, dizziness, vertigo, nausea. Cognition remained stable over time, as tested utilizing the Cogstate® tests battery. No reports of psychosis were reported. Overall, this long-term extension study corroborates the findings of its parent trials and validates ESK efficacy, safety, and tolerability in TRD pts., and MDD pts. at suicidal risk.
Canuso et al. (2018) #NCT02133001 [16]	Phase II, DB, RCT: To examine the efficacy of standard-of-care Tx plus intranasal ESK vs. placebo for rapid decrease of major depressive disorder symptoms, including suicidality, in pts. at imminent risk of suicide.	In a proof-of-concept study, 68 pts. were randomized to receive ESK (84 mg) or placebo twice weekly for four weeks in addition to standard-of-care therapy. The primary endpoint to test efficacy was the MADRS score change four hours after the initial dose. Secondary objectives included a suicide risk assessment via the SIBAT: measured at 24 hours and on the 25th day.	At 4 hours, LS means difference = -5.3. At 24 hours, LS means difference = -7.2. The MADRS score enhanced significantly more in the ESK group than in the placebo group, but not at day 25 where the LS means difference = -4.5. The MADRS score reduction was greater in the ESK group. after four hours, but not at 24 hours or day 25. Reductions in clinician global judgments of suicide risk ratings did not differ between groups.	Despite day 25 data, these preliminary findings suggest that the addition of intranasal ESK with comprehensive standard-of-care Tx may result in a far rapid rate of improvement of depressive symptoms, most importantly including measures of suicidal ideation, in depressed pts. at imminent risk for suicide.
Fu et al. (2020) #NCT03039192 [17]	Phase III, DB-RCT: To evaluate ESK on a patient with MDD exhibiting suicidal ideation and intent	A total of 226 MDD pts. meeting the screening criteria were randomized to 84 mg of ESK or a placebo twice weekly for four weeks, along with standard-of-care Tx (i.e., psychiatric hospitalization and OAD)	ESK improved MADRS scores both at four and 24 hours after Tx and outperformed the placebo group. LS mean difference: -3.8. However, suicide severity did not differ across groups during monitoring.	ESK proved to be efficacious in diminishing depressive symptoms in MDD pts. at imminent risk for suicide (i.e., exhibiting suicidal ideation and intent)
Ionescu et al. (2020) #NCT03097133 [18]	Phase III, DBL-RCT: To evaluate ESK on a patient with MDD exhibiting suicidal ideation and intent	A total of 227 received ESK and were included in efficacy/safety analyses; 184 (80.0%) completed DB Tx.	Significant improvement in MADRS total score was seen in the ESK group of average of -15.7 vs placebo -12.4, both with standard-of-care Tx, at 24 hours. A secondary endpoint was seen in a significant reduction in CGISS-r scores.	ESK proved to be efficacious in diminishing depressive symptoms in MDD pts. at imminent risk for suicide (i.e., exhibiting suicidal ideation and intent)
Canuso et al. (2021) #NCT03039192, #NCT03097133 [19]	Post-hoc analysis/pooled data: To outline a pair of identically conducted, double-blinded phase 3 studies, following which ESK nasal spray was approved by many authorities for the indication of TRD in MDD pts. with high suicidal risk.	Across the ASPIRE (I & II) studies, 456 pts. received standard-of-care therapy (hospital stay new OAD) with either ESK-84 mg or placebo. The administration took place two times a week for four weeks.	MADRS change from baseline to 24 hours was the primary endpoint. The secondary endpoint was a change in CGISS-r scores. Both endpoints were analyzed using ANCOVA. The pooled averaged showed a reduction of 3.8 pts MADRS score in the ESK group vs. placebo control. The between-group differences were negligible	Improvements were seen at four hrs. and maintained throughout the four-week trial period. The ASPIRE I & II trials were the basis for ESK’s supplemental approval by the FDA (and other organizations) for approved indication in MDD pts at imminent risk for suicide.
Singh et al. (2016) #NCT01640080 [20]	DB-RCT to assess the efficacy, safety, and dose response of an IV ESK infusion in pts. with TRD.	This trial was conducted utilizing 30 TRD pts. Pts. were randomly assigned 1:1:1 to either receive an IV infusion of 0.20 mg/kg, 0.40 mg/kg ESK, or placebo over 40 minutes on day 1.	Of 30 pts., 29 of them completed the study. The LS mean difference from baseline to day two in MADRS score for ESK of 0.20 mg/kg and 0.40 mg/kg dose groups was (SE) -16.8 (3.00) and -16.9 (2.61), respectively, and showed significant improvement compared with placebo =23.8 {2.97).	A rapid onset of robust antidepressant effects was observed in pts. with TRD after a 40-minute IV infusion of either 0.20 mg/kg or 0.40 mg/kg of ESK. Measures for depressive symptoms didn’t differ significantly between the two doses of IV ESK, suggesting that ESK may allow for better tolerability while maintaining efficacy at even lower dosages.
Correia-Melo et al. (2020) [21]	DB-RCT to compare ESK and ketamine. (non-inferiority margin of 20%)	Participants received ESK 0.25 mg/kg or ketamine 0.5 mg/kg via single IV infusion for 40 minutes to compare depression remission rates 24 hours after the intervention, as measured by the primary endpoint in MADRS score change.	In the study, 29 participants received ketamine, and 34 received ESK. After 24 hrs., 24.1% of the ketamine group and 29.4% of the ESK group showed remission	The study confirmed non-inferiority and in fact, an improvement in 5.3% of higher remission rate. The efficacy, safety, and tolerability of ESK and ketamine were comparable for treating TRD within 24 hours post-tx.
Targum et al. (2019) #NCT01998958 [22]	To test the reliability of remote-based raters vs. site-based raters as functional unblinding due to TEAEs may conflate findings of double-blind, placebo-controlled studies.	Audio-digital recordings of site-based MADRS interviews were obtained from a subset of pts. during a double-blind, placebo-controlled study (SYNAPSE trial) examining ESK nasal spray vs. placebo in TRD pts..	None of the seven placebo-assigned pts. achieved a tx response or remission at the 2-hour post-dose assessment. Four of the seven ESK-assigned pts. (57.1%) achieved a tx response at two-hr. post-dose, and 3 pts. (42.9%) achieved remission.	The remote site-independent raters practically duplicated the site-based MADRS score reductions in yielding a 92.9% predictive value for matching tx response and remission rates. This study also displays that blinded remote ratings (without the possibility of functional unblinding) are comparable to site-based ratings of the efficacy of ESK nasal spray.
Morrison et al. (2018) #NCT02094378 [23]	DB-RCT, two-period crossover study to evaluate intranasal ESK's effect on healthy participants' cognitive functioning.	Twenty-four participants aged 19–49 years were randomized to either ESK 84 mg vs. placebo.	ESK was associated with substantial cognitive performance impairment at 40 min post-dose for all five Cogstate® tests compared to one-hour pre-dose.	A single dose of ESK (84mg) was associated with cognitive decline in mental performance. Cognitive performance returned to placebo-comparable levels after two hours post-dose. Future testing is deemed necessary to discover the appropriate dosing and recovery times involved with the effects of ESK on cognition and mental task performance.
Van De Loo et al. (2017) [24]	DB-RCT to evaluate the effect of a single dose of intranasal ESK (84 mg) compared to a placebo on on-road driving performance.	Twenty-six healthy volunteers aged 21 to 60 years were enrolled in this study. Participants conducted the standardized 100-km on-road driving test 8 hours after tx administration.	Twenty-four participants completed the study. No significant SDLP difference was found between ESK and placebo (p = 0.7638), whereas the SDLP after mirtazapine was significantly higher when compared to placebo (p = 0.0001).	No substantial differences in driving performance were observed eight hours. Overall, administration of intranasal ESK did not significantly impair driving performance 8 hours after tx. Oral mirtazapine (30mg), however, significantly impaired road driving performance.

Efficacy in treatment-resistant-depression and FDA approval

ESK was developed by Janssen Pharmaceutical's Research and Development branch (JRD) as a nasal-spray formulation (Spravato®), which was considered an innovative method, with the FDA giving the drug a “breakthrough designation.” This special designation allows for an accelerated protocol to have a drug approved [4,25]. The FDA drew upon the data of five phase III trials, including TRANSFORM 1-3 and SUSTAIN 1 and 2 [10-14]. The three TRANSFORM trials were to determine short-term efficacy, while SUSTAIN-1 was a withdrawal study to examine the maintenance of effect, and SUSTAIN-2 was a long-term study aimed at determining ESK's safety parameters [10-14,25]. The most pivotal trials for FDA approval were TRANSFORM-2 and SUSTAIN-1 [4,11,13]. In 2020, the FDA approved an additional indication for ESK to treat MDD patients with suicidal ideation exhibiting clear intent, putting them at risk of imminent suicide, according to the ASPIRE I and 2 trials [4,17,18].

JRD initiated three identically structured short-term Phase III DB-RCT's to test intranasal ESK's efficacy [10-12]. The TRANSFORM trials also assessed safety, but the primary focus was on determining the ability of ESK to promote anti-depressive effects in combination with an OAD when compared to an OAD and placebo alone [10-12]. Where TRANSFORM-1 and 2 examined ESK in adult patients between the ages of 18 and 64 [10,11], TRANSFORM-3 enrolled individuals ≥65 [12]. Although all three studies resulted in a meaningful reduction in the total Montgomery-Asberg Depression Rating Scale (MADRS) score, TRANSFORM-2 was the most influential short-term efficacy trial. The FDA used TRANSFORM-2 to make its decision as it met the pre-defined statistical significance cut-off that TRANSFORM 1 and 3 did not [10-12].

For all the TRANSFORM trials, eligible patients consisted of patients with recurrent MDD (per the Diagnostic and Statistical Manual of Mental Disorders) or those who had a single episode of MDD (≥2 years) without psychotic features, as confirmed by the Mini-International Neuropsychiatric Interview (MINI) [10-12]. During these trials, the administration of ESK (during a 4-week randomized, placebo-controlled phase) occurred after a 4-week period of screening and observation. Throughout this phase, patients continued taking the same OADs to measure if there was a lack of improvement (defined as ⩽25% improvement in the MADRS total score) [10-12,25]. A MADRS total score ≥28 at weeks two and four for TRANSFORM 1 and 2 and a MADRS score ≥24 in TRANSFORM-3 were respectively required to enter randomization [10-12]. It is important to note that many other scales were used, such as the Clinical Global Impression-Severity (CGI-S) by investigators to judge the reductive degree of depressive symptoms; the Patient Health Questionnaire 9-item (PHQ-9) to rate symptoms of depression; the Sheehan Disability Scale (SDS) for patients to rate study impact on socio-occupational disability; the Generalized Anxiety Disorder 7-item (GAD-7) Scale to measure anxiety; and the EuroQol-5 dimension-5 level (EQ-5D-5L) to rate overall health measures [10-12]. For the sake of conciseness, the review has limited the discussion to only the primary and secondary endpoints.

TRANSFORM-1 resulted in no statistical significance with ESK 84 mg/OAD compared with antidepressant/placebo (least squares [LS] means difference [95% CI]: -3.2 [-6.88, 0.45]; 2-sided p-value = .088). ESK 56 mg/OAD could not be formally tested, but the LS mean difference was -4.1 [-7.67, -0.49] (nominal 2-sided p-value = .027).

In TRANSFORM 3, the primary endpoint estimate was −3.6 within a 95% CI (−7.20, 0.07); in patients aged 65-74, the adjusted mean difference between treatment groups was -4.9, with a 95% CI of -8.96 to -0.89, implying their symptoms improved statistically. However, in older adults ≥75, the adjustment equaled -0.4 (-10.38, 9.50) and lacked statistical significance. For those who developed depression before 55, the adjusted mean average was -6.1 (-10.33, -1.81), also indicating a statistically significant improvement in depressive symptoms [12]. The adjusted mean difference was 3.1 (-4.51, 10.80) for those who developed depression after 55, again lacking statistical significance [12]. Significantly, in TRANSFORM-3, a higher percentage of older patients in the ESK group achieved both clinical response (23.6% versus 12.3%) and clinical remission (15.3% versus 6.2%) compared to those who received placebo [12,25]. However, as seen above, the age of onset of depression was an important variable, and in patients with a younger age of onset of depression, ESK yielded more beneficial results [12].

TRANSFORM-2 was the most important in terms of FDA approval and short-term efficacy results. It consisted of four phases, the first of which was a prospective four-week screening and observation phase during which patients were given their current OAD and the treatment response was assessed, as described above, only after which ESK dosing was undertaken by the study group [11]. In the second phase, patients received a new OAD alongside either ESK or placebo nasal sprays, respectively [11]. The third phase encompassed a post-treatment follow-up at 24 weeks [11]. The difference in LS means between the ESK 84 mg/OAD vs. placebo control group was -3.2, with a 95% CI ranging from -6.88 to 0.45 and a nominal two-sided p-value of 0.088 [11]. To evaluate ESK 56 mg, statistical significance for ESK 84 mg was required, resulting in an inadequate evaluation of ESK 56 mg. The LS mean difference for ESK 56 mg was -4.1 [11].This study took place across a multitude of international cities across the span of two years; at day 28, a far greater reduction in MADRS scores was seen than with placebo and OAD alone (LS means difference = -4.0, SE=1.69, 95% confidence interval (CI)= -7.31, -0.64), supporting the efficacy of ESK nasal spray as a quick-acting antidepressant at both 56 mg and 84 mg [11].

Efficacy in MDD with suicidal ideation

In 2020, the FDA importantly approved a supplemental New Drug Application (sNDA) for ESK, for which it was approved for treating MDD patients at high risk of suicide [4,17-19,25]. Canuso et al. (2018) published an innovative phase II DB-RCT in which JRD researchers enrolled 68 participants in a proof-of-concept trial to test intranasal ESK's efficacy in MDD patients exhibiting suicidal ideation and intent [16]. Over four weeks, participants were randomized to receive either ESK (84 mg) or a placebo twice weekly, alongside standard-of-care treatment involving initial psychiatric hospitalization, and the introduction or modification of an OAD was provided to all participants. At four hours, LS means difference = -5.3, standard error (SE) = 2.10; (effect size =0.61); and at 24 hours, LS means difference = -7.2, SE = 2.85; (effect size = 0.65), with the MADRS score enhancing significantly more in the ESK group than in the placebo group, except at day 25, where the LS means difference = -4.5, SE = 3.14; (effect size=0.35) [16]. The MADRS score reduction was greater in the ESK group after four hours (impact size=0.67) but not at 24 hours (effect size=0.35) or day 25 (effect size=0.25) [16]. Reductions in clinician global judgments of suicide risk ratings did not differ between groups [16]. However, the LS mean difference after 25 hours was -4.5 with an effect size of 0.35 and was not considered significant. ESK, nonetheless, displayed the ability to considerably reduce depressive symptoms after four hours [16]. This was a crucial finding with important implications considering the potential in an MDD patient with suicidal ideation and the intent to take their own life in the early hours of a depressive bout. The study validated the need for larger-scale trials to assess ESK benefits in suicidal MDD patients [16].

Subsequently, JRD conducted the ASPIRE I and II clinical trials, which investigated and confirmed the benefits of ESK in MDD patients at imminent risk of suicide [17,18]. Both were phase III trials instead of much larger sample sizes. Between June of 2017 and December of 2018, ASPIRE I enrolled 226 participants with active suicidal ideation who needed to be hospitalized [17]. A larger reduction in MADRS scores was observed in the ESK control group, alongside standard-of-care at 24 hours (LS mean difference [SE]: -3.8 [1.39]; 95% CI, -6.56 to -1.09; 2-sided p = .006), as well as at earlier time points (four-hour mark) and later periods in the trial during the four-week double-blind treatment [17]. ASPIRE II had an identical setup and reached similar findings: of 227 who received ESK and were included in efficacy/safety analyses, 184, or 80.0%, of them completed treatment [18]. Significant improvements in MADRS total scores in the ESK group were on average -15.7 (standard deviation [SD]=11.56), while the placebo was -12.4 (SD=10.43), alongside standard of care, at 24 hours (LS mean difference [SE]: -3.9 [1.39], 95% CI: -6.60, -1.11; 2-sided p = .006) [18]. This was also noted at the four-hour mark and at later points in the study. Moreover, researchers employed a key secondary endpoint by measuring the Clinical Global Impression of Severity of Suicidality-revised (CGISS-r) score, which saw rapid reductions in both ASPIRE I and II [17,18]. Canuso et al. (2021) published pooled data for both of these trials, which is referenced in Table 2 [18]. Combining both trials for a total of 456 MDD patients at greater risk of suicide, the average MADRS score reduction was calculated to be statistically significant (LS mean difference = -3.8, 2-sided p=0.006) [17-19].

Moreover, using patients from TRANSFORM 1 and 2, SUSTAN-1 was a long-term phase 3 withdrawal study of 705 participants [13]. By utilizing a withdrawal approach in which the active therapy is withdrawn, and patients are continued on the drug of choice vs. placebo, the amount of time spent on the placebo can be minimized [25]. This allows for a healthy evaluation, as was the case in SUSTAIN-1 of the efficacy of ESK plus OAD vs. placebo + OAD in delaying the recurrence of depressive symptoms in TRD patients, by making the primary efficacy endpoint of this study the result in stable remitters and stable responders [13]. ESK adjunctively given with an OAD significantly delayed the relapse rate compared to the placebo control group at statistical significance (p=0.003) [13]. ESK alongside OAD treatment decreased the risk of relapse by 51% (hazard ratio (HR)=0.49; 95% CI, 0.29-0.84) among patients who were in stable remission and 70% (HR, 0.30; 95% CI, 0.16-0.55) among stable responders compared to the control group on OAD and placebo alone [13]. 

Comparison to ketamine

Ketamine has been approved by the FDA for anesthetic purposes since 1970, and the concept of NMDA antagonists having anti-depressive properties is not a recent phenomenon [5,25]. Berman et al. (2000) originally reported on MDD patients who responded quickly and strongly to a single intravenous (IV) infusion of ketamine in a very small study of seven patients using .05mg/kg [25,26]. 

Of note, Singh et al. (2016) published the outcomes of a DB-RCT where IV ketamine was tested independently for its efficacy and safety in TRD [27]. Using a cohort of 67 patients with TRD aged between 18 and 64 and 68 patients as a control, both groups were randomized to an administration of either IV ketamine (0.5 mg/kg of body weight) or IV placebo over 40 minutes, two or three times weekly for up to four weeks, the last two weeks being an optional open-label double-blinded phase for patients who didn't achieve a response in the first 15 days [27]. Ultimately, the MADRS score changes to day 15, the primary efficacy endpoint, saw significant reductions in the ketamine cohort compared to the placebo group [27]. The mean difference in MADRS score on the 15th day in the ketamine cohort treated twice a week was -18.4 (SD=12.0) compared to -5.7 (SD=10.2) for placebo [27]. When ketamine was given three times a week, MADRS change was -17.7 (SD=7.3) vs. -3.1 (SD=5.7) for placebo [27]. During the optional open-label phase, comparable observations were observed for ketamine, as measured on day five [27]. Importantly, the dose and both dosing frequencies were generally well tolerated; although patients experienced TEAEs, some experienced dissociative effects that subsided after repeated treatments [27].

There has been a recent trend of prescribing ketamine off-label to treat patients with TRD in the past decade [25]. Ketamine, however, has a substantial propensity for serious side effects, the most concerning of which are its psychotomimetic (i.e., dissociative symptoms), neurotoxic, and cognitively impairing effects that make it impractical for common use in clinical practice [25]. In the same year, Singh et al. (2016) published the efficacy and safety outcomes of a DB-RCT to explore the dose response of IV ESK infusion in patients with TRD [20]. Researchers enrolled 30 patients with TRD to compare a single IV infusion of 0.2 or 0.4 mg/kg of ESK vs. placebo [20]. The primary endpoint was the difference in MADRS total score between the first and second days [20]. Non-responsive patients on the first day were randomly assigned to IV ESK on day four [20]. Just two days after treatment, patients treated with ESK at both doses showed greater clinical improvements, as seen by a total MADRS score reduction compared to placebo alone [20]. ESK started working within two hours and showed a strong anti-depressive response. TEAEs did occur, with headache, dizziness, and nausea being the most reported [20].

However, the latter effects were described as mild, generally tolerable, and transient. Also, the tendency for side effects was greater with a higher dosage, but the anti-depressive effects were relatively similar between dosages, indicating ESK could be used at low doses and still produce the desired effect [20]. 

As IV ketamine and IV ESK had both been independently investigated, researchers wanted to further determine whether ESK was at least as good as ketamine. In alignment with this interest, 63 patients with TRD were enrolled in a DB-RCT, non-inferiority trial to directly compare the efficacy and safety of ESK and ketamine in treating TRD [21]. Correia-Melo et al. (2020) compared both drugs, administering a 40-min-single IV infusion of racemic ketamine and ESK at 0.25mg/kg and 0.5 mg/kg, respectively [21]. Based on MADRS scores, there was a greater rate of remission after 24 hours in the ESK cohort than those receiving ketamine in 5.3% of the patients [21]. As a result, not only did ESK show an absence of inferiority, but it also demonstrated superiority to ketamine in its ability to combat TRD at very low doses with quick onset, establishing itself as a more practical alternative for clinical usage [21].

Safety

ESK's most common side effects in the TRANSFORM trial program were nausea, feeling distant, dizziness, and headache, while other clinical research shows similar outcomes [10-12]. SUSTAIN-2 was a longer-term, phase III study aimed at evaluating ESK and antidepressant safety/tolerability in patients with TRD [14]. In this study, TEAEs occurred in 723, or 90.1%, of patients. However, only 55 (6.9%) were categorized as having suffered severe effects, including lacunar stroke, hypothermia, heavy sedation, confusion, suicidal ideation, or partial seizures [14]. For the most part, the intranasal route of administration has been well-received through ESK trials, as it is hypothesized to not lead to blood concentrations as rapidly as the IV route [25]. Importantly, SUSTAIN-2 also demonstrated, at a 48-week follow-up, that ESK had maintained its antidepressive effect [14].

SUSTAIN-3 was a five-year safety study to analyze the long-term effects of ESK [15]. The authors published the interim results, outlining the safety profile of ESK with intermittent dosing for up to 4.5 years in SUSTAIN-3, comprising an extensive 2,769 cumulative patient years [15]. The findings were consistent with earlier studies. TEAEs were reported to be mild and transient (headache, dizziness, vertigo, and nausea being the most common) [15]. No reports of psychosis were reported [15]. Overall, this long-term extension study corroborates the findings of its parent trials and validates ESK efficacy, safety, and tolerability in TRD patients and MDD patients at suicidal risk. 

Among the problematic side effects, researchers found that ESK could raise blood pressure by 7-12mmHG [4]. Hence, providers have been warned to take precautions in patients with arterial diseases, such as a history of aneurysms or arteriovenous malformations, which are contraindications to treatment [4]. The addictive potential of ESK is also worrisome, which is why mitigation strategies such as Spravato's REMS program have been put in place [4]. Patient monitoring for patients using ESK is crucial and should be regular and ongoing throughout their time on the drug [4]. Taste changes, vertigo, dizziness, and dissociation were common in the SUSTAIN trials [13-15]. However, these effects were categorized as mild to moderate and subsided after a couple of hours. A testament to the tolerability of ESK can also be seen in the fact that only seven percent of individuals quit treatment due to adverse effects [28].

Reliability study (to rule out functional unblinding)

Despite double-blinding and randomization, it is common for patients to undergo "functional unblinding" where psychedelic drugs are being tested, as patients may be able to recognize through their symptomatology that they have been administered a substance of some kind and not a placebo. To make sure that ESK nasal spray trial findings were valid and would further produce reliable results, a comparability study was performed using remote or site-independent and site-based raters through the use of audio and digital-based recordings [22]. In conclusion, remote raters practically duplicated site-based findings in MADRS score reductions, yielding a 92.9% predictive value for matching treatment responses and remission rates [22]. This study indicates that blinded remote ratings (without the possibility of functional unblinding) are comparable to site-based ratings concerning the efficacy of ESK nasal spray [22].

Impact on cognition

Throughout all the trials of ESK nasal spray thus far, cognition has also been addressed as to how or whether ESK affects cognition. In SUSTAIN-3, cognition remained intact throughout a five-year post-market surveillance study [15], as judged over time through the use of the Cogstate® test battery. In two earlier published Phase I DB-RCTs, cognition was directly tested [23,24]. In a DB-crossover study, Morrison et al. (2018) administered 84mg ESK and the Cogstate® test battery to a cohort of 24 healthy adults [23]. The study group performed worse on all of the tasks in the test battery compared to one hour before treatment [23]. A single dose of ESK (84mg) was associated with a decline in mental performance but returned to placebo-comparable levels after two hours post-dose [23]. In a different DB-RCT, Van de Loo et al. (2017) evaluated 26 healthy young volunteers driving performance after administering ESK and mirtazapine vs. placebo and judged their driving performance on a 100km road test eight hours after administration [24]. No meaningful difference was found between performances after eight hours [24], reinforcing the notion that ESK has primarily transient effects that subside after a given interval. 

Route of administration and potency benefits

The non-inferiority study above compared IV infusions of racemic ketamine and ESK, but one of the hallmark benefits of ESK is the intranasal formulation that comprises its route of administration [28]. The intranasal route is generally more convenient than the intravenous route due to the rapid onset of action, increased bioavailability, and high systemic absorption via the nasal mucosa [28]. Young et al. (2019) demonstrated that intranasal ESK effectively yields plasma levels comparable to IV drug infusions [29]. Wang et al. (2021) performed a large-scale meta-analysis and highlighted how evidence shows that multiple administrations of intranasal ESK reduce the rate and intensity of psychotomimetic effects as well as have reduced cardiac effects [30]. Moreover, with a four-fold greater affinity for the NMDA receptor than the R-enantiomer, ESK can potently exert its effect at relatively low doses and maintain it for a long period, making it both more efficacious and safer to use [21,28].

Approved therapies

Until the approval of ESK as an adjunct for its use in TRD, the only pharmacological therapy approved for TRD had been Olanzapine supplemented with Fluoxetine (Prozac®) [25]. The time for this regimen to work has been a limiting factor in its efficacy [25,28]. According to the Cleveland Clinic, the FDA currently approves four medications for the treatment of TRD, in addition to ESK, for a total of five [31]. These include aripiprazole (Abilify®) and brexpiprazole (Rexulti®), which are third-generation antipsychotic medications [30]. Additionally, quetiapine (Seroquel®) and olanzapine (Zyprexa®), second-generation antipsychotic medications, are also now being used [31]. Similar to ESK, Quetiapine is approved only as an adjunct treatment alongside market OADs for the treatment of TRD [31]. The FDA has also approved electroconvulsive therapy (ECT), a procedure that involves passing an electric current through your brain, causing a short seizure that may stimulate nerve signals and exert brain changes that can improve mood [31]. Repetitive transcranial magnetic stimulation (rTMS), a noninvasive therapy, has also been approved for TRD, which utilizes a magnetic coil to influence the brain’s natural electrical activity with the ability to enhance mood [31].

It is worth mentioning here that ESK was recently compared to extended-release quetiapine in an open-label, single-blind, Phase 3b active-controlled trial where ESK was compared to extended-release quetiapine [32]. Researchers assigned patients, in a 1:1 ratio, to receive flexible doses of ESK nasal spray or extended-release quetiapine, both being used in combination with an SSRI or SNRI [32]. The primary end point was remission (a score of ten or less on the MADRS) at week eight, while the key secondary end point was no relapse through week 32 after remission at week eight [32]. After 336 patients were assigned to the ESK group and 340 to the quetiapine group, more patients in the ESK group went into remission at week eight than the quetiapine group (91 of 336 patients [27.1%] vs. 60 of 340 patients [17.6%]; P=0.003) [32]. Moreover, more participants in the ESK group showed no relapse through 32 weeks after achieving remission at week eight (73 of 336 patients [21.7%] vs. 48 of 340 patients [14.1%]) [32]. Post-follow-up results after 32 weeks, the percentage of patients with remission, the percentage of patients with a treatment response, and the change in the MADRS score from baseline favored esketamine nasal spray [32].

Developing research

Alternatively, there is new research underway regarding both treatments and routes of administration. For example, a study was conducted where ESK may be delivered using hydrogel-forming microneedle arrays via a transdermal patch [33]. This method could be used as a solution to some of the delivery drawbacks of using an IV or nasal spray. Additionally, this could be a more affordable option compared to IVs and possibly reduce the addiction factor of the nasal spray [33]. Overall, for some patients, this minimally invasive transdermal patch could provide a more comfortable and less stressful approach to receiving the treatment [33]. However, the downside to this study's therapeutic course is the time to onset of effect that it may take for the transdermal patch to exert its full effect [33]. Although this has only been tested on rats, researchers plan on continuing this method using a larger animal model [33].

The exploration of biomarkers and receptors linked to ESK therapy is a subject of great interest. Ketamine and ESK are engaging prospects to produce swift antidepressant effects in TRD due to their novel mechanisms of action, but they are still not fully understood [7,8,34]. The general agreement in the literature is that ketamine and ESK both affect the α-Amino-3-hydroxy-5-methyl-4-isoxazolepropionic acid (AMPA) receptor through its agonistic effect on glutamate, causing a downstream increase in BDNF [34,35]. Other relevant biomarkers have been thought to include, but are not limited to, alterations in pro-inflammatory cytokines interleukins (IL) 1, IL-6, IL-10, vascular endothelial growth factor (VEGF), tumor necrosis factor-alpha (TNF-α), as well as interferon-gamma (IFN-γ). Other receptors include the mu, kappa, and delta-opioid receptors, respectively [34,35]. The decrease in the expression of BDNF, a member of the neurotrophic protein family, is believed to display some type of causal relationship with neuropsychiatric disorders. BDNF levels decrease in MDD and increase after antidepressant treatment, and similarly with ketamine and ESK [34,35].

Importantly, biomarkers are capable of being quantified in the peripheral blood, but it is unclear whether peripheral blood concentrations correlate with CNS concentrations [35], an understanding of which could lead to revolutionary outcomes. A comprehensive systematic review and meta-analysis was conducted of 460 blood markers, but in conclusion, the results were unable to find any consistency in peripheral blood markers other than BDNF, which showed elevated levels after ketamine and, by default, ESK use [35]. A deeper understanding and identification of which biomarkers are affected and how may eventually unveil the full mechanism of ketamine and ESK, or their metabolites, or potentially shed light on the underlying pathophysiologic mechanisms behind diseases as well. Larger and more frequent studies are needed to facilitate this research, but this knowledge could lead to more guided pharmacological therapy for ketamine or ESK, as well as other antidepressants. Moreover, a large number of experimental therapies are currently being evaluated, as well as many investigations into the biochemistry behind TRD. New and potential agents to treat TRD can be classified as drugs that work on the HPA axis, metalonergic pathways, dopaminergic pathways, cytokine-mediated pathways, non-steroidal anti-inflammatory drugs (NSAIDs), neuromodulation techniques, and alternative therapies (i.e., yoga, meditation, etc.) [36].

Limitations and future perspectives

As this narrative review does not comprise a systematic review, there may be studies and additional trials that have not been included. There is a scarcity of ESK studies involving patients with co-existing mental illnesses, as well as studies in adolescents, in whom ESK use is not currently approved [4]. The WHO estimates that one in seven individuals between the ages of 10 and 19 suffers from a mental illness, accounting for 13% of the global disease burden, with depression and suicide being among the leading causes of disability and death in this group, respectively [37]. This warrants further research on the potential benefits of ESK use in adolescent populations. Similarly, statistically significant data is lacking for the effectiveness of ESK in adults over 75 [12]. Alongside such quantitative variables, ESK research specific to qualitative factors such as race and sex needs further exploration. Although the outcomes of a single five-year safety and tolerability ESK study have been included [15], most of the trials conducted and outlined in this review are short-term studies, which limits the ability to evaluate ESK treatment in the long term, another area where further research is indicated. 

More broadly speaking, the lack of a universally recognized definition of TRD poses a challenge to accurately detecting the prevalence of TRD, impeding developments in fundamental and translational research [3]. The absence of a proper classification for TRD leads to heterogeneity in the decision-making process and order of treatments from both a public policy and clinical standpoint [3]. A more accurate and reliable description of TRD may promote more innovative and precision-guided therapies [3].

## Conclusions

When used in combination with an OAD, ESK demonstrates itself as a beneficial supplement in the treatment of TRD and MDD with acute suicidal intent. Its novel mechanism of action and intranasal formulation provide its own benefits, making it a more practical alternative to more invasive therapies such as IV ketamine. The side effect profile of ESK seems generally mild and tolerable, with patients mainly suffering from transient effects. Results from ESK's five-year safety study have not highlighted any new concerns, and the large number of patient trial completions has reinforced ESK's reputation for being relatively safe and tolerable in clinical usage. However, further research is still required, and ESK's adverse effects should not be overlooked, as they still warrant the need for ESK use to be performed under clinical supervision.

Notably, the fundamental research leading to ESK's FDA approval for both its indications utilized altering levels of heterogeneous patient populations and the exclusion of patient populations with co-existing mental illnesses. This can tend to produce variable effect sizes and raise questions about its generalizability. There remains a scarcity of ESK research across multiple domains and considerable room for expansion in this regard towards more narrowed quantitative and qualitative research, as well as investigations in patients with co-existing mental illnesses. A crucial need exists to fully understand the neuropharmacology behind how ESK exerts its effects and what molecular components are involved. A universally accepted definition of TRD and more focused investigations can help facilitate more precision guided ESK use and promote the discovery of further comparative benefits, some of which have already emerged in the academic literature. In the interim, intranasal ESK as an adjunctive therapy to OADs has shown itself to be an efficacious therapy that meets an urgent need for patients with TRD and MDD with suicidal intent.
